# Draft Genome Sequence of *Lactobacillus salivarius* L28 Isolated from Ground Beef

**DOI:** 10.1128/genomeA.00955-17

**Published:** 2017-09-28

**Authors:** Diana I. Ayala, Peter W. Cook, David L. Campos, Mindy M. Brashears, Henk den Bakker, Kendra K. Nightingale

**Affiliations:** International Center for Food Industry Excellence, Department of Animal and Food Sciences, Texas Tech University, Lubbock, Texas, USA

## Abstract

In this report, we describe the draft genome sequence of a newly discovered probiotic strain, *Lactobacillus salivarius* L28. *L. salivarius* L28 demonstrates antagonistic effects against human foodborne pathogens, including *Escherichia coli* O157:H7, *Salmonella* spp., and *Listeria monocytogenes*, in coculture experiments and food matrices.

## GENOME ANNOUNCEMENT

Probiotics are live, naturally occurring bacteria or fungi that, when administered in adequate amounts, benefit the host by improving microbial balance (1, 2). The use of probiotics has increased in recent years due to their well-known health-promoting effects and their potential to replace subtherapeutic antibiotics in livestock (3–6). Probiotics containing *Lactobacillus* spp. have been reported to reduce the prevalence of foodborne pathogens in feces and hides and decrease the colonization of peripheral lymph nodes by* Salmonella* spp. in livestock (6–8). Probiotics benefit the host by producing antimicrobial compounds (i.e., bacteriocins and organic acids), competing for epithelial receptors and nutrients in the gastrointestinal tract, producing enzymes and vitamins, and improving the intestinal barrier and homeostasis (9). *Lactobacillus* spp. have been commonly isolated from plants, silage, raw meat, fermented foods, oral cavities, and gastrointestinal tracts of humans and animals.

*Lactobacillus salivarius* is a bacteriocin-producing bacterium that has been identified as a promising probiotic due to its ability to modulate gut microbiota. They enhance immune response and reduce host colonization by pathogenic bacteria, thus increasing animal performance (10, 11). *L. salivarius* L28 was isolated from ground beef; preliminary experiments show that L28 reduces *Escherichia coli* O157:H7, *Salmonella* spp., and *Listeria monocytogenes* by 4.5, 6.5, and 8.5 log_10_ CFU/ml, respectively, compared to controls cultivated without L28 (our unpublished data). Because *L. salivarius* L28 demonstrated the potential to control foodborne pathogens *in vitro*, we sequenced the L28 genome to gain further insight into antagonistic mechanisms and identify genetic markers unique to L28.

*L. salivarius* L28 was cultivated in MRS broth, and genomic DNA was isolated using the Invitrogen Purelink DNA extraction kit (Thermo Fisher Scientific, Waltham, MA, USA). Pure genomic DNA was used as input material for library preparation with NexteraXT version 2.0 (Illumina, Inc., San Diego, CA, USA). DNA libraries were paired-end sequenced using a 2 × 250-bp V2 kit on an Illumina MiSeq platform. Raw reads were trimmed using Trimmomatic version 0.33 (12) and assembled using SPAdes version 3.5 (13). The L28 draft genome was annotated using the NCBI Prokaryotic Genome Annotation Pipeline (14). Reads were assembled into 96 contiguous sequences (contigs). The size of the L28 draft genome was estimated at 2,028,405 bp with an average G+C content of 32.7%; the longest contig was 275,535 bp, the *N*_50_ value was 79,042 bp, and the genome coverage was 118×. A total of 1,982 coding sequences, 64 tRNAs, and 31 rRNAs were predicted. Two prepeptides, or inducing factors for bacteriocin synthesis, were identified in the general annotation, two incomplete prophages were identified by PHASTER (15), and one potential plasmid (99,553 bp) was identified. Additionally, 12 potential virulence factors with nucleotide identities greater than 72% were found when compared to the virulence factors database (VFDB) (16), and one gene for potential tetracycline resistance with 90% nucleotide identity was identified when compared to the comprehensive antibiotic resistance database (CARD) (17).

### Accession number(s).

This whole-genome shotgun sequencing project has been deposited in DDBJ/ENA/GenBank under the accession no. NDYW00000000. The version described in this paper is the first version, NDYW01000000.
